# Supplementary material for: Detection and characterization of ESBL-producing Escherichia coli and additional co-existence with mcr genes from river water in northern Thailand
Source: PeerJ. 2022 Nov 14;10:e14408. doi: 10.7717/peerj.14408 (PMC9671034; doi:10.7717/peerj.14408)
Supplement: Supplemental Information 1 — The filtered reads were used as an input for Unicycler, a genome assembly program. Quast was used to evaluate draft genome assemblies by computing various metrics shown below. [file peerj-10-14408-s001.docx]

**Supplementary Table S1.** Assembly Statistics of all ten ESBL-producing *E. coli* isolated from the Kok River and the Kham River, Chiang Rai. Thailand. The filtered reads were used as an input for Unicycler, a genome assembly program. Quast was used to evaluate draft genome assemblies by computing various metrics shown below.

| Assembly | EH1201 | EH1307 | EH2102 | EH2301 | EH9101 | EK1201 | EK2303 | EK2501 | EK2504 | EK9101 |
| --- | --- | --- | --- | --- | --- | --- | --- | --- | --- | --- |
| # contigs (>= 0 bp) | 204 | 135 | 236 | 146 | 231 | 120 | 192 | 99 | 96 | 338 |
| # contigs (>= 1000 bp) | 95 | 86 | 98 | 112 | 121 | 67 | 100 | 51 | 51 | 116 |
| Total length (>= 0 bp) | 5072849 | 5371212 | 5253142 | 4710161 | 5122679 | 4969314 | 5078150 | 4914550 | 4911613 | 538836 |
| Total length (>= 1000 bp) | 5019682 | 5348406 | 5185990 | 4691865 | 5067444 | 4945724 | 5038257 | 4891383 | 4891267 | 5311223 |
| # contigs | 141 | 106 | 158 | 128 | 169 | 83 | 124 | 71 | 67 | 220 |
| Largest contiges | 688203 | 382891 | 511965 | 292732 | 480852 | 464210 | 358499 | 410045 | 402826 | 472699 |
| Total length | 5053924 | 5362091 | 5226920 | 4703796 | 5102806 | 4957784 | 5056209 | 4906071 | 4902811 | 5348880 |
| GC (%) | 50.79 | 50.72 | 50.74 | 50.59 | 50.55 | 50.74 | 50.66 | 50.59 | 50.58 | 50.38 |
| N50 | 224191 | 190675 | 190854 | 93436 | 169321 | 223904 | 154435 | 241870 | 241870 | 123708 |
| N75 | 87898 | 86578 | 111554 | 46819 | 73433 | 133903 | 87066 | 102743 | 104716 | 43789 |
| L50 | 8 | 10 | 9 | 17 | 11 | 8 | 12 | 8 | 8 | 14 |
| L75 | 17 | 19 | 17 | 34 | 22 | 15 | 23 | 15 | 15 | 33 |
| # N’s per 100 kbp | 0 | 0 | 0 | 0 | 0 | 0 | 0 | 0 | 0 | 0 |

All statistics are based on contigs of size >= 500 bp

Given a set of contigs, the *N50* is defined as the sequence length of the shortest contig at 50% of the total genome length.

Given a set of contigs, each with its own length, the *L50* is defined as the count of the smallest number of contigs whose length sum makes up half of the genome size.
