# Supplementary material for: Detection and characterization of ESBL-producing Escherichia coli and additional co-existence with mcr genes from river water in northern Thailand
Source: PeerJ. 2022 Nov 14;10:e14408. doi: 10.7717/peerj.14408 (PMC9671034; doi:10.7717/peerj.14408)
Supplement: Supplemental Information 2 [file peerj-10-14408-s002.docx]

**Supplemental Table S1**. Antibiogram profile of *E. coli* isolates from river water in northern Thailand.

| Pattern | Antibiogram profile | No. isolates | | Number of resistant ABTs |
| --- | --- | --- | --- | --- |
|  |  | Kok River | Kham River |  |
| 1 | AMC-AMP-Cip-C-S-CN-MEM-TE-SXT-FEP-CAZ-CTX | 1 | 0 | 12 |
| 2 | AMC-AMP-Cip-NA-C-S-CN-TE-SXT-FEP-CAZ-CTX | 1 | 0 | 12 |
| 3 | AMC-AMP-Cip-NA-C-S-CN-TE-FEP-CAZ-CTX | 0 | 1 | 11 |
| 4 | AMC-AMP-Cip-NA-C-S-TE-SXT-FEP-CAZ-CTX | 1 | 0 | 11 |
| 5 | AMC-AMP-Cip-NA-CN-TE-SXT-FOX-FEP-CAZ-CTX | 1 | 0 | 11 |
| 6 | AMC-AMP-NA-C-S-ETP-TE-SXT-FOX-FEP-CTX | 0 | 1 | 11 |
| 7 | AMP-Cip-NA-C-S-CN-TE-SXT-FEP-CAZ-CTX | 1 | 0 | 11 |
| 8 | AMC-AMP-Cip-NA-S-TE-SXT-FEP-CAZ-CTX | 1 | 0 | 10 |
| 9 | AMC-AMP-Cip-C-S-TE-SXT-FEP-CAZ-CTX | 0 | 2 | 10 |
| 10 | AMC-AMP-Cip-C-TE-SXT-FEP-CAZ-CTX | 0 | 1 | 9 |
| 11 | AMC-AMP-Cip-NA-S-TE-SXT-FEP-CTX | 1 | 0 | 9 |
| 12 | AMC-AMP-NA-C-TE-SXT-FOX-FEP-CTX | 0 | 1 | 9 |
| 13 | AMP-Cip-NA-C-S-CN-TE-FEP-CTX | 2 | 0 | 9 |
| 14 | AMP-Cip-NA-S-CN-STX-FEP-CAZ-CTX | 0 | 1 | 9 |
| 15 | AMP-Cip-NA-S-TE-STX-FEP-CAZ-CTX | 1 | 3 | 9 |
| 16 | AMC-AMP-Cip-NA-C-S-MEM-TE | 0 | 1 | 8 |
| 17 | AMC-AMP-C-S-CN-FEP-CAZ-CTX | 0 | 1 | 8 |
| 18 | AMC-AMP-Cip-S-TE-SXT-FEP-CTX | 1 | 0 | 8 |
| 19 | AMP-C-S-CN-TE-FEP-CAZ-CTX | 0 | 1 | 8 |
| 20 | AMP-Cip-NA-TE-SXT-FEP-CAZ-CTX | 1 | 0 | 8 |
| 21 | AMP-Cip-NA-S-CN-TE-FEP-CTX | 1 | 0 | 8 |
| 22 | AMC-AMP-Cip-C-S-TE-SXT | 0 | 1 | 7 |
| 23 | AMC-AMP-Cip-NA-C-S-TE | 0 | 1 | 7 |
| 24 | AMC-AMP-Cip-TE-FEP-CAZ-CTX | 1 | 0 | 7 |
| 25 | AMP-Cip-NA-C-S-CN-TE | 1 | 0 | 7 |
| 26 | AMP-Cip-NA-C-S-TE-SXT | 1 | 0 | 7 |
| 27 | AMP-Cip-NA-S-TE-FEP-CTX | 0 | 1 | 7 |
| 28 | AMC-AMP-Cip-NA-C-S-TE | 0 | 0 | 7 |
| 29 | AMP-S-TE-SXT-FEP-CAZ-CTX | 1 | 0 | 7 |
| 30 | AMC-AMP-C-S-CN-TE | 1 | 0 | 6 |
| 31 | AMC-AMP-Cip-C-S-TE | 1 | 0 | 6 |
| 32 | AMC-AMP-Cip-FEP-CAZ-CTX | 1 | 0 | 6 |
| 33 | AMC-AMP-Cip-NA-S-TE | 0 | 1 | 6 |
| 34 | AMC-AMP-TE-FEP-CAZ-CTX | 0 | 1 | 6 |
| 35 | AMP-S-TE-SXT-CAZ-CTX | 1 | 0 | 6 |
| 36 | AMP-C-CN-FEP-CAZ-CTX | 0 | 1 | 6 |
| 37 | AMP-C-TE-FEP-CAZ-CTX | 0 | 1 | 6 |
| 38 | AMP-Cip-NA-S-TE-CAZ | 1 | 0 | 6 |
| 39 | AMP-Cip-NA-CN-FEP-CTX | 1 | 0 | 0 |
| 40 | AMC-AMP-Cip-FEP-CTX | 1 | 0 | 5 |
| 41 | AMC-AMP-Cip-NA-TE | 0 | 1 | 5 |
| 42 | AMC-AMP-Cip-S-TE | 0 | 1 | 5 |
| 43 | AMC-AMP-C-S-STX | 0 | 2 | 5 |
| 44 | AMC-AMP-C-S-TE | 0 | 1 | 5 |
| 45 | AMC-AMP-FEP-CAZ-CTX | 0 | 1 | 5 |
| 46 | AMC-AMP-S-TE-STX | 1 | 1 | 5 |
| 47 | AMP-C-CN-FEP-CTX | 0 | 1 | 5 |
| 48 | AMP-S-TE-SXT-CAZ | 1 | 0 | 5 |
| 49 | AMP-TE-FEP-CAZ-CTX | 1 | 0 | 5 |
| 50 | AMP-CN-MEM-FEP-CTX | 1 | 0 | 5 |
| 51 | AMP-Cip-FEP-CAZ-CTX | 1 | 0 | 5 |
| 52 | AMP-Cip-NA-S-TE | 1 | 1 | 5 |
| 53 | Cip-NA-C-TE-SXT | 0 | 1 | 5 |
| 54 | AMC-AMP-Cip-NA | 0 | 1 | 4 |
| 55 | AMC-AMP-Cip-TE | 1 | 0 | 4 |
| 56 | AMC-AMP-TE-STX | 0 | 1 | 4 |
| 57 | AMP-FEP-CAZ-CTX | 0 | 1 | 4 |
| 58 | AMP-Cip-FEP-CTX | 1 | 0 | 4 |
| 59 | AMP-Cip-NA-CTX | 1 | 0 | 4 |
| 60 | AMP-Cip-S-TE | 1 | 0 | 4 |
| 61 | AMP-NA-S-TE | 0 | 1 | 4 |
| 62 | AMP-S-TE-SXT | 1 | 0 | 4 |
| 63 | AMP-TE-FEP-CTX | 1 | 0 | 4 |
| 64 | AMC-AMP-S | 0 | 3 | 3 |
| 65 | AMC-AMP-TE | 1 | 3 | 3 |
| 66 | AMP-NA-S | 0 | 1 | 3 |
| 67 | AMP-S-TE | 0 | 2 | 3 |
| 68 | NA-S-TE | 0 | 1 | 3 |
| 69 | AMC-AMP | 2 | 4 | 2 |
| 70 | AMP-NA | 1 | 0 | 2 |
| 71 | AMP-TE | 6 | 4 | 2 |
| 72 | S-TE | 0 | 2 | 2 |
| 73 | TE-SXT | 1 | 0 | 2 |
| 74 | AMC | 1 | 2 | 1 |
| 75 | AMP | 7 | 20 | 1 |
| 76 | Cip | 1 | 0 | 1 |
| 77 | MEM | 1 | 0 | 1 |
| 78 | S | 1 | 1 | 1 |
| 79 | SXT | 1 | 0 | 1 |
| 80 | TE | 3 | 3 | 1 |

Cip, ciprofloxacin; NA, Nalidixic acid; C, chloramphenicol; S, streptomycin; CN, gentamicin; MEM, meropenem; ETP, ertapenem; TE, tetracycline; CT, colistin; AMC, amoxicillin-clavulanic acid; AMP, ampicillin; STX; trimethoprim/sulfamethoxazole; FOX; cefoxitin, FEP; cefepime, CAZ; ceftaxidime, และ CTX; cefotaxime.
