# Supplementary material for: Detection and characterization of ESBL-producing Escherichia coli and additional co-existence with mcr genes from river water in northern Thailand
Source: PeerJ. 2022 Nov 14;10:e14408. doi: 10.7717/peerj.14408 (PMC9671034; doi:10.7717/peerj.14408)
Supplement: Supplemental Information 3 [file peerj-10-14408-s003.docx]

**Supplemental Table S3**. The top 25 discriminatory refinement loci among ten *E. coli* genomes used for constructing canonical wgMLST tree

| **#  Number** | **Splits** | **Gene_name** | **Occ(%)** | **Annotation** |
| --- | --- | --- | --- | --- |
| 1 | 3 | yqjF | 100 | Inner membrane protein YqjF |
| 2 | 5 | group_1235 | 100 | hypothetical protein |
| 3 | 2 | rimP | 100 | Ribosome maturation factor RimP |
| 4 | 5 | potF_2 | 100 | Putrescine-binding periplasmic protein precursor |
| 5 | 5 | group_707 | 100 | hypothetical protein |
| 6 | 1 | rplS | 100 | 50S ribosomal protein L19 |
| 7 | 4 | trmJ | 100 | tRNA (cytidine/uridine-2'-O-)-methyltransferase TrmJ |
| 8 | 1 | tatA | 100 | Sec-independent protein translocase protein TatA |
| 9 | 1 | rplT | 100 | 50S ribosomal protein L20 |
| 10 | 1 | clpS | 100 | ATP-dependent Clp protease adapter protein ClpS |
| 11 | 4 | metJ | 100 | Met repressor |
| 12 | 2 | rnk | 100 | Regulator of nucleoside diphosphate kinase |
| 13 | 2 | group_7622 | 100 | hypothetical protein |
| 14 | 1 | group_7644 | 100 | hypothetical protein |
| 15 | 3 | metK | 100 | S-adenosylmethionine synthase |
| 16 | 2 | ybgC | 100 | Acyl-CoA thioester hydrolase YbgC |
| 17 | 3 | slyA | 100 | Transcriptional regulator SlyA |
| 18 | 4 | group_8527 | 100 | Aminodeoxyfutalosine deaminase |
| 19 | 5 | pyrD | 100 | Dihydroorotate dehydrogenase (quinone) |
| 20 | 3 | group_8966 | 100 | PspA/IM30 family protein |
| 21 | 4 | apaH | 100 | Bis(5'-nucleosyl)-tetraphosphatase [symmetrical] |
| 22 | 2 | group_9472 | 100 | hypothetical protein |
| 23 | 3 | group_9641 | 100 | hypothetical protein |
| 24 | 4 | ubiI | 100 | 2-octaprenylphenol hydroxylase |
| 25 | 5 | bioH | 100 | Pimeloyl-[acyl-carrier protein] methyl ester esterase |
